# Envisioning public health as a learning health system

**DOI:** 10.1002/lrh2.10465

**Published:** 2024-10-21

**Authors:** Theresa A. Cullen, Lisa Villarroel

**Affiliations:** ^1^ Pima County Health Department Tucson Arizona USA; ^2^ Arizona Department of Health Services Phoenix Arizona USA

This Special Issue of *Learning Health Systems* seeks to understand what it would take for public health to become a learning health system. The selected articles highlight the required organizational insights and foundational components, such as including public health partners in care networks and ensuring timely, relevant public health data in cycles of public health learning—both of which reflect the foundational public health core functions of Assessment, Assurance, and Policy.^1^

The transition to a learning public health system may herald the next phase of public health. Public Health 1.0 envisioned governmental entities providing functions to improve public health during a time of growth of clinical and public healthcare. Public Health 2.0, as outlined in the 1988 Institute of Medicine's *The Future of Public Health*,^2^ focused on traditional public health agency programs. In 2016, Public Health 3.0 stressed multi‐partner engagement around social determinants of health.^3^

We propose that Public Health 4.0 integrate historical lessons from public health with those from a learning healthcare system to embody a Learning Public Health System model.^4^ By expanding stakeholders, integrating organizational learning into our processes, continually using data and evaluation to form new public health practices, and incorporating self‐evaluation and communication transparency, public health can continually learn and improve.

As public health officials in state and local health departments, we acknowledge that our own institutions are not yet learning public health systems. Our foundational cycles of Assessment, Assurance, and Policy often buckle due to the lack of workforce, funding, and infrastructure. However, we believe that aligning with a learning health system framework would recommit public health to rapid cycle innovation and response as we face stubborn foes like heat, loneliness, substance use, and vaccine hesitancy.

This published collection of articles helps inform the framework of a learning health system that needs to be envisioned and actualized.

One approach for the creation of a learning public health system model is to broaden the conceptual framework of what is included in a learning health system. Rather than insulating the model within a healthcare system, participating partners would include public health and community‐based organizations. The case study from Semprini et al.^5^ presents how a rural cancer network worked with the public health cancer registry to access their data to enhance patient outcomes. Along a similar model, Meigs et al.^6^ propose incorporating community‐based organizations (CBOs) into a learning health system at all stages, with examples of successful integrations in refugee initiatives. These papers illustrate the expansion of learning health systems beyond previously defined boundaries, resulting in improved health outcomes.

These authors show that breaking open the learning health system to include other partners is itself possible and essential. In the future, a rural cancer network could seamlessly share patient outcomes with public health entities; public health would work with healthcare systems and the rural community‐based organizations to enhance education, prevention, earlier access to cancer care, and evaluate the impact of these interventions as well as outcomes.

Public health can also create its own learning health system: a Learning Public Health System (LPHS) as conceived by Tenenbaum^4^ and exemplified by Wolfenden et al.^7^ in a chronic disease prevention model. To strengthen public health data in such an LPHS, Guralnik^8^ proposes standardizing electronic heatlh record (EHR)‐based public health surveillance by repurposing already established computable phenotypes and data platforms, while Rajamani et al.^9^ details how data systems can be enhanced with an academic partnership with public health. To strengthen public health policy, Tenenbaum^4^ suggests that an LPHS would leverage data and take into account the demographics, climate, and politics of a region to make recommendations. Villegas‐Diaz et al.^10^ specify the inclusion of environmental privacy‐securing data and Kilbourne et al.^11^ suggest a framework to address evidence‐based policymaking. To strengthen public health evaluation, Brennan and Abimbola^12^ posit that After Action Reports (AARs), utilized by public health for emergency management documentation, could be repurposed as a learning tool. The public health function of case investigation and contact tracing in outbreaks would benefit from this type of ongoing evaluation, utilizing process and metrics that have been proposed by the 7‐1‐7 Alliance.^13^

One may imagine the positive impact that a learning public health system would have had during a pandemic. With EHR‐based public health surveillance, public health would have rapid and just‐in‐time information about healthcare system capacity and disease states. Academic partners would help public health with aggregating, analyzing, and displaying the data to ensure transparency. Community organizations at the table would help to inform public health actions and policies that align with the needs and politics of a region. AARs would be used to ensure ongoing improvement to public health surveillance and assurance.

Reimagining the entire learning health system framework into a Health and Human Services Learning Health System, as Tenenbaum^4^ suggests, may be the best option—a large‐scale, full partnered set of constant learning cycles to understand and guide the complex issues impacting health and health status. The “blurring of boundaries across domains like medicine, public health, and social services” acknowledges that health is not driven by clinical medicine but by determinants of health including economics, housing, climate, and culture.

The complexity of issues impacting population health and wellness requires that the role and functions of public health are reimagined and reconstructed. Despite the constraints on public health resources, public health remains uniquely positioned to tackle the multifactorial problems impacting our population. Rapid‐cycle iterations are essential, as we saw during the COVID‐19 response, to help inform, guide, and transform not only public health, but the health of the nation.

Of course, any attempt to broaden the scope of learning health systems will encounter barriers. New partnerships between public health and healthcare systems may be difficult to create given the historical divide between these sectors. Sustained commitment of community leaders, individuals, family, schools, and business engagement as well as bureaucratic support are required to create this new model of learning public health systems. Agreements on data access and data governance as well as prioritization and sharing of obstacles and lessons learned between systems is critical. Creating a shared vision of community wellness and health, supported by learning, needs to occur.

Rajamani et al proposes that becoming a learning health system will be “a journey.” There is no immediate switch that can create, adjust, or overhaul the current public health and healthcare framework. The first step of this journey, we believe, is to make new and learning relationships with ourselves, our neighbors, our communities, and healthcare. Academics, meet and integrate your local public health department in your work. Cancer registries, seek out your rural hospital cancer network and the rural public health department. Hospital Chief Medical Officers, meet your public health counterparts. Public health departments, reach within and out, and set the table where everyone has a seat and a voice.

Once there, talk about a learning public health system—you may be surprised at how we can learn together.
